# Arthur David Griffiths

**Published:** 1905-09

**Authors:** 


### Obituary Notices.

### ARTHUR DAVID GRIFFITHS,
M.D. Brux., Bridgend.

Dr. A. D. Griffiths was educated at Weston-super-Mare and at Wycliffe College, Stonehouse. On matriculating at the London University, he entered University College, Bristol, passing his Preliminary Scientific before entering the Medical Department. At the Bristol General Hospital he obtained the Martyn Memorial and Saunders Scholarships. He then studied for awhile at King's College Hospital, London; and after passing the Conjoint Board in 1894, he assisted Drs. Rose and Carless in getting out their *Manual of Surgery*, many of the illustrations being drawn by his hand.

He then settled in the Forest of Dean, but sold his practice in 1897 to enter on a course of study in Brussels, where he obtained the M.D. of the University. He afterwards practised four years in Ramsgate, but eventually settled at Bridgend, Glamorganshire, where he held a number of important appointments, including that of medical referee to the Coal Owners' Association, and where he was building up a large practice.

The circumstances of his death were peculiarly sad and painful, not only on account of its appalling suddenness from heart failure, but also that it should occur so soon after his success in completely vindicating his character at the Swansea Assizes last December after a foul attempt at blackmail and slander by a patient, when he was awarded £500 damages. The strain and stress of this dreadful time undoubtedly undermined his naturally vigorous health, which was further affected by the rush of work entailed in combating the serious epidemic of typhoid fever which soon afterwards developed in his neighbourhood, and also by his anxiety concerning his wife, who had contracted the disease, their only child being very ill also.

At eleven p.m. on the night of his death (June 15th) he held a long conversation on the telephone with his brother in Bristol, who interrogated him as to his own health. He answered that he felt tired after such heavy days, and that he thought his heart must be a bit weak as he experienced difficulty in walking. He agreed to his brother's suggestion of assistance, and retired to rest in his usual good spirits. At 4.30 a.m. he went to the door of his wife's room and called the nurse, complaining of agonising pain and putting his hand to his heart. She gave a stimulant, and telephoned at once to his friend, Dr. Thomas, who, though arriving with all speed, pronounced life to be extinct.

Dr. Griffiths was a skilful diagnostician and an expert in electrotherapy; he brought to bear on his work, in which he always rejoiced, a mind of considerable originality. Very popular among his fellow-students, he was still more so among his brother practitioners and his patients, and by his personal charm and joyous nature won the love and admiration of all his many friends. Upright, courteous and brave, full of promise and hope, his early death, at the age of 34, comes with a very poignant sense of loss to all who knew him, and casts a deep gloom on the neighbourhood in which he lived.

